# CCR2 dependent recruited pro-inflammatory monocytes contribute to the development of left ventricular hypertrophy in mice upon transverse aortic constriction

**DOI:** 10.1371/journal.pone.0318407

**Published:** 2025-04-21

**Authors:** Lars Eichhorn, Jan Lukas Kleiner, Benedikt Bartsch, Mariam Louis Fathy Nazir, Yunyang Zhang, Mark Coburn, Stilla Frede, Christina Katharina Weisheit

**Affiliations:** 1 Department of Anesthesiology and Intensive Care Medicine, University Hospital Bonn, Bonn, Germany; 2 Department of Anesthesiology, Helios Klinikum Bonn/Rhein-Sieg, Bonn, Germany; 3 Heart Center Bonn, Department of Medicine II, University Hospital Bonn, Bonn, Germany; 4 Department of Anesthesiology and Intensive Care Medicine, Faculty of Medicine, University of Cologne, University Hospital Cologne, Cologne, Germany; Pennsylvania State University Hershey Medical Center, UNITED STATES OF AMERICA

## Abstract

C-C chemokine receptor type 2 positive monocytes are recruited from the circulation to infiltrate inflamed tissue. Left ventricular (LV) hypertrophy caused by pressure overload presents with a chronic myocardial inflammation in our mouse model of transverse aortic constriction (TAC). Recent analyses demonstrated that deficiency of fractalkine receptor CX3CR1 leads to a pro-inflammatory phenotype characterized by increased numbers of Ly6C^high^ macrophages in the myocardium due to chemokine receptor CCR2 dependent monocyte recruitment from the circulation. Here, we analyzed the role of CCR2 in the development of left ventricular hypertrophy using Ccr2^-/-^ mice. We were able to show that a lack of CCR2 dependent recruited Ly6C^high^ monocytes in the myocardium reveled cardioprotective effects resulting in less hypertrophy and reduced brain natriuretic peptide (BNP) expression, as biomarker of heart failure, in the myocardium. CCR2-deficiency caused an increase in neutrophil and a reduced macrophage accumulation in the myocardium in response to pressure overload. The cytokine pattern measured in the LV tissue indicates a significantly reduced release of IL1-β whereas TNF-α concentrations are increased following TAC. IL-6 secretion is not altered by the lack of CCR2 and the pro-remodeling cytokine IL-10 is not increased either. This study highlights the importance of CCR2 in the pathogenesis of LV hypertrophy and the relevance of CCR2 dependent recruited monocytes for the orchestration of the cardiac immune response.

## Introduction

Left ventricular (LV) hypertrophy serves as an indicator of organ impairment in hypertension, correlating with heightened morbidity and mortality rates [1]. Arterial hypertension-induced LV hypertrophy represents a complex cardiac condition originating from the responses of both myocyte and non-myocyte components to mechanical and neuro-humoral stimuli [2]. In reaction to chemokines, immune cells move from the bloodstream to the heart, subsequently releasing pro-hypertrophic and pro-inflammatory cytokines such as TNF-α, IL-1β, and IL-6 [3].

In the realm of cardiovascular disease, the mobilization and infiltration of peripheral monocytes into affected tissues are predominantly viewed as maladaptive responses due to their association with unfavorable outcomes, including infarct expansion, left ventricular (LV) systolic dysfunction, LV dilation, and progression of atherosclerotic plaques [4]. Recent research has highlighted the significance of C-C chemokine receptor type 2 (CCR2) expression in facilitating the recruitment of bone marrow (BM)-derived Ly6C^high^ monocytes into inflamed tissues [5] and the provenience of CCR2^+^ cardiac macrophages that are derived from hematopoietic progenitors. We could already show that in LV hypertrophy CCR2^+^ macrophages are likely to be expanded through CCL2 dependent monocyte recruitment [6].

When CCL2 binds to the CCR2 receptor, it induces a structural change in the receptor, which subsequently activates the associated G-protein, driving intracellular signaling pathways, including the inhibition of adenylyl cyclase, activation of phosphatidylinositol 3-kinase (PI3K), and modulation of small GTPases [7]. These signaling mechanisms lead to the activation of proteins necessary for cell motility and the organization of actin filaments, enabling cellular movement along a chemokine gradient. Together, these processes are crucial for mediating immune responses and inflammatory activity [8].

Correspondingly, interventions targeting monocyte recruitment by disrupting MCP-1/CCL2 (monocyte chemoattractant protein 1) and CCR2 signaling have shown promise in mitigating excessive inflammation and conferring protection in mouse models of myocardial infarction and atherosclerosis [9]. Consequently, CCR2 inhibitors are currently being investigated as potential therapeutic agents for cardiovascular diseases [10,11]. Additionally, prior studies by ourselves and others have identified functionally distinct subsets of macrophages residing within the myocardium, particularly under steady-state and pressure-overload conditions [12].

The identification of distinct cardiac macrophage populations aligns with the evolving body of literature exploring the origins of tissue-resident macrophages in various tissues and organs. In 2007, Nahrendorf and colleagues illustrated the sequential recruitment of Ly6C^high^ and Ly6C^low^ monocytes to the heart following MI, with CCR2 and CX3CR1 being the respective mediators [13]. CCR2^−^-macrophages colonize the heart during embryonic and early postnatal stages, persisting independently of peripheral monocyte influx under normal conditions. They play pivotal roles in promoting coronary development, facilitating cardiac regeneration, and supporting electrical conduction within the atrioventricular node [14]. Our recent publication revealed that cardiac macrophages do not undergo local proliferation in response to pressure overload-induced left ventricular (LV) hypertrophy in our model; rather, they seem to be recruited to the site of myocardial inflammation via CCL2 chemotaxis [6]. Moreover, by blocking CX3CR1-CXCL1 chemotaxis, we observed a shift in the immune response phenotype toward a pro-inflammatory pattern, marked by increased accumulation of pro-inflammatory cytokines, neutrophils, and CCR2^+^ inflammatory macrophages, resulting in cardioprotection.

This project aims to investigate whether the observed cardioprotective effect can be attributed to the enhanced recruitment of inflammatory monocytes from the circulation to the myocardium via CCL2-dependent mechanisms.

## Materials and methods

### Mice

Female C57BL/6J mice, aged between 10 and 14 weeks, were either obtained from Charles River or bred in the central animal facilities of the Medical Faculty at Bonn University (House of Experimental Therapy, HET). The Ccr2^-/-^ mice were sourced from Jackson Laboratory (strain designation: B6.129S4-Ccr2tm1Ifc/J; stock number: 004999) and subsequently bred in the same central facilities at Bonn University. Prior to inclusion in experiments, all mice underwent a minimum of eight backcrosses to the C57BL/6J background. The mice were maintained in a specific pathogen-free environment, housed in isolated, ventilated cages with unrestricted access to food and water. All animal procedures were sanctioned by the ethics board at the Ministry of Nature, Environment and Consumer Protection of North Rhine-Westphalia (LANUV Recklinghausen; permit numbers: 84–02.04.2011.A313, 84–02.04.2016.A374) and were conducted under the supervision of the central animal facilities at Bonn University (HET, Venusberg-Campus 1, Bonn, Germany). Surgical procedures were performed under anesthesia and analgesia, adhering to protocols aimed at minimizing animal suffering.

### Transverse aortic constriction (TAC)

**Fig 1:**
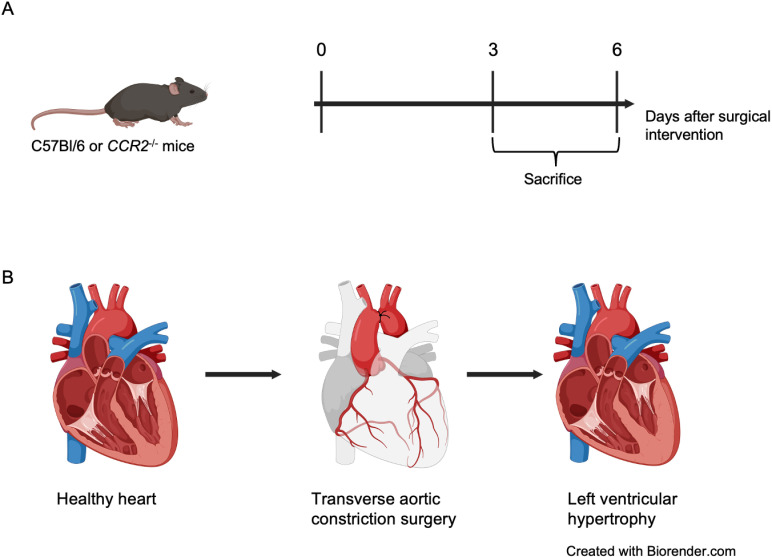
Experimental protocol and TAC intervention. **Created in BioRender. Rathmann, S. (2024) BioRender.com/u27q473.** **(A)** C57Bl/6 mice (Wt) or *Ccr2*^*-/-*^ mice underwent TAC or sham surgery. At 3 and 6 days after surgical intervention animals were sacrificed. **(B)** Transverse aortic constriction induces left ventricular hypertrophy due to pressure overload.

A closed-chest approach was employed to reduce surgical trauma, with the intervention being performed as minimally invasively as possible [6,15]. The standardized experimental protocol is depicted in Fig 1. Mice were anesthetized using isoflurane (2% vol), intubated with a 1.2 mm OD cannula, and ventilated at a rate of 150 breaths per minute with tidal volumes set to 8–10 mL/kg body weight using a small animal ventilator (Harvard Apparatus; Holliston, MA). A 27 G spacer was utilized to standardize the aortic constriction. Sham-operated controls underwent identical procedures, except for the tightening of the suture around the aorta. For pain management post-surgery, buprenorphine was administered subcutaneously at a dose of 0.1 µg/g every 8 hours for three days. Mice were euthanized at 3 and 6 days post-surgery via atlanto-occipital dislocation. The selected time points for analysis were based on preliminary results, reflecting the immune response phases following TAC, including early and late inflammatory phases as well as the remodeling phase [6,12].

### Determination of heart-weight/body-weight-index

Prior to euthanasia, the body weight (BW) of each mouse was recorded. The heart weight (HW) was measured after careful extraction of the heart from the thoracic cavity, ensuring thorough rinsing with PBS to eliminate residual blood. The HW/BW index was calculated in mg HW per g BW.

### Flow cytometry

Immune cell isolation from blood and left ventricular (LV) tissue was conducted as previously described. Peripheral blood (100 µl) was collected in EDTA-coated tubes (Sigma Aldrich, St. Louis, MO) and subjected to erythrocyte lysis using RBC lysis buffer (Thermo Fisher Scientific, Waltham, MA). Whole hearts were perfused with PBS, minced using a single-edged blade, and digested in RPMI medium containing 1 mg/ml collagenase-2 and 1 mg/ml DNase I (Sigma Aldrich) at 37°C for 60 minutes. To prevent nonspecific Fc receptor binding, cells were incubated with anti-mouse CD16/32 (BD Biosciences, Franklin Lakes, NJ) for 10 minutes at 4°C. Subsequent staining with fluorochrome-conjugated antibodies was performed in the dark for 20 minutes at 4°C, utilizing antibody clones from Thermo Fisher, BD Biosciences, and BioLegend: CD45 (AFS98), F4/80 (BM-8), CD115 (c-fms), Ly6C (HK1.4), Ly6G (1A8), and LIVE/DEAD Fixable Dead Cell Stain Kit (Thermo Fisher). Absolute cell counts were determined by adding a fixed number of CaliBRITE APC beads (6 µm size) (BD Biosciences) as an internal reference. Additionally, the LV tissue and spleen weights were recorded prior to digestion, and cell numbers were normalized to 100 mg of tissue. Flow cytometry was executed on a BD LSR Fortessa II, with data analysis performed using FlowJo software (BD Biosciences).

### Real time PCR

LV tissue samples were preserved at -80°C until homogenization in Trizol, following the manufacturer’s guidelines (Thermo Fisher). Reverse transcription of 2000 ng total RNA into cDNA was conducted using the High Capacity cDNA Reverse Transcription Kit (Thermo Fisher). The expressions of BNP (Mm01255770_g1, amplicon length 68) and 18S (Mm02601777_g1, amplicon length 76) were quantified via qPCR employing the TaqMan PCR detection system (Life Technologies, Carlsbad, CA). The 18S ribosomal RNA served as an internal control. The quantification of specific cDNA was normalized to 18S using the Δct method, with differences between treatment groups calculated via the DDct method. Results are presented as 2-ΔΔct values relative to the specified controls [16,17].

### Cytokine and chemokine measurement

For the analysis of cytokines and chemokines, LV tissue samples were stored at -80°C until homogenization in RIPA buffer. The analysis was conducted following the manufacturer’s protocol. Quantikine ELISA kits (R&D Systems, Wiesbaden, Germany) were utilized for the detection of TNF-α, IL-6, IL-1β, and IL-10. The concentrations of cytokines and chemokines were normalized to 100 µg of total protein, measured using a BCA Protein Assay Kit (Thermo Fisher) in accordance with the manufacturer’s instructions.

### Statistical analysis

Prior to conducting statistical tests, the assumptions regarding data distribution (e.g., normality and homogeneity of variance) were assessed. The number of experiments and mice per group is detailed in the figure legends. Comparisons between two groups were performed using Student’s t-tests, while one-way or two-way analyses of variance, followed by Tukey’s test for multiple comparisons, were applied as appropriate. Statistical analysis was executed using Prism 8 (GraphPad Software, Inc., La Jolla, CA). Results are presented as mean ± SD unless otherwise specified; a p-value of less than 0.05 was deemed statistically significant.

## Results

**Fig 2:**
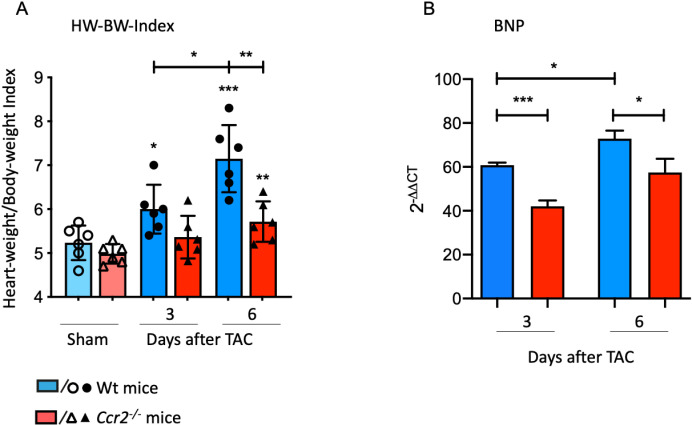
CCR2 expression modulates the development of LV hypertrophy in response to pressure overload. **(A)** Heart-Weight/Body-Weight-Index of Wt and *Ccr2*^*-/-*^ mice calculated 3 and 6 days after transverse aortic constriction (TAC) and sham operation. **(B)** BNP mRNA expression pattern in LV tissue determined 3 and 6 days after TAC in Wt and *Ccr2*^*-/-*^ mice. * above individual columns indicate significant differences between TAC and respective sham group; *P <0.05, **P<0.01, *** P<0.001, ****p < 0.0001.

Our recent findings strongly suggest a CCL2-dependent recruitment of monocytes to the myocardium in response to pressure overload in the absence of the chemokine receptor CX3CR1 [6]. To investigate the specific role of the chemokine receptor CCR2 in the development of left ventricular hypertrophy (LVH), *Ccr2*^*-/-*^ mice were exposed to a minimally invasive mouse model of TAC, and the results were compared to those of C57BL/6 (Wt) animals [15]. The Heart-Weight/Body-Weight-Index (HW/BW-Index) was calculated to assess the progression of cardiac hypertrophy. Myocardial hypertrophy serves as a cardioprotective mechanism in response to pressure overload. When compared to CCR2 competent mice (Wt), *Ccr2*^*-/-*^ mice exhibited significantly less hypertrophy than the respective Wt groups. In *Ccr2*^*-/-*^ mice, the HW-BW-Index showed a significant increase at day 6 after TAC compared to sham (Fig 2A). Conversely, we noted a significant rise in HW-BW-Index in Wt animals at day 3 and 6 compared to sham. The lesser degree of hypertrophy development is reflected by the BNP expression of the LV tissue. BNP serves as a well-accepted biomarker for diagnosis of heart failure [18]. Here, we observe significantly higher BNP RNA expression in Wt animals at day 3 and 6 after TAC compared to *Ccr2*^*-/-*^ mice (Fig 2B).

These results indicate that CCR2 deficiency leads to a slower and less pronounced progression of LV hypertrophy development.

### CCR2 deficiency caused an increase in neutrophil and a reduced macrophage accumulation in the myocardium

**Fig 3:**
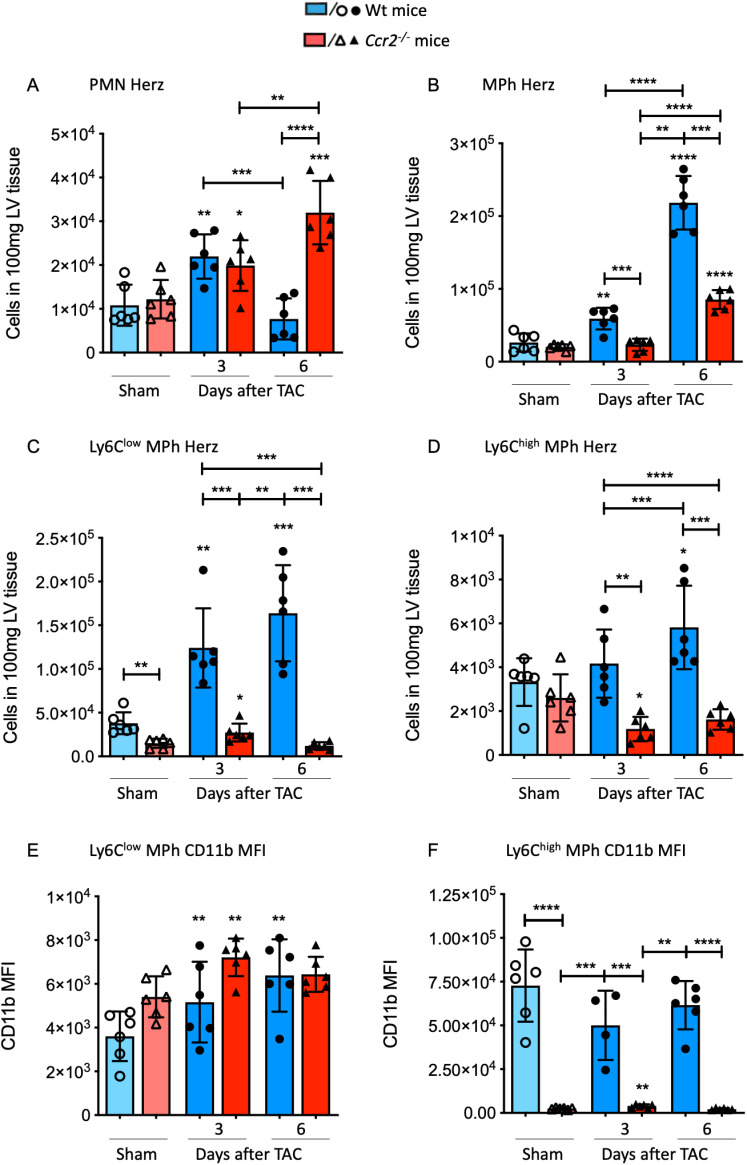
CCR2 deficiency influences cellular immune response during chronic pressure overload. **(A)** Number of neutrophils (PMN), (B) macrophages (MPh) and macrophage subpopulations (C, **D)** Ly6C^low^ and Ly6C^high^ macrophages in the LV tissue was determined by flow cytometry analysis in *Ccr2*^*-/-*^ mice and Wt mice 3 and 6 days after TAC and sham operation. The particular cell subsets were defined as follows: neutrophils as CD45^+^ F4/80^-^ Ly6G^+,^ macrophages as CD45^+^ F4/80^+^ Ly6G^-^; Ly6C^high^ and Ly6C^low^ macrophages were further discriminated according to the respective Ly6C surface expression; (E, **F)** Determination of the mean fluorescence intensity (MFI) of CD11b on the surface of Ly6C^high^ and Ly6C^low^ macrophages. * above individual columns indicate significant differences between TAC and respective sham group; *P <0.05, **P<0.01, *** P<0.001, ****p < 0.0001.

The time-points for immune response analysis were selected based on recent findings, which emphasized a notable accumulation of major histocompatibility complex class II (MHC-II) positive cells within the initial week following TAC intervention, showcasing the impact of pressure overload [12]. To investigate the role of CCR2 expression in orchestrating the cardiac immune response, we examined the immune reaction in *Ccr2*^*-/-*^ mice subjected to pressure overload. Subsequent to TAC intervention, we noted a marked rise in myocardial neutrophil counts at day 3 compared to sham in both groups (Fig 2A). While in Wt mice, this elevation was transient, with neutrophil numbers returning to sham levels by day 6 after TAC, in *Ccr2*^*-/-*^ mice, neutrophil numbers continued to rise until day 6 (Fig 3A). Conversely, a reverse trend was observed in the macrophage population. We observed a notable increase in cell numbers at day 3 in Wt mice compared to sham and CCR2-deficient mice. Although macrophage numbers continued to rise significantly in Wt animals until day 6 post-TAC, this response was considerably attenuated in *Ccr2*^*-/-*^ mice (Fig 3B). Specifically, The number of macrophages was 2.5 times higher in Wt animals than in CCR2-deficient mice.

Considering that the macrophage group is heterogeneous and that at least two subpopulations have been implicated in our recent studies, we investigated the kinetics of Ly6C^low^ CCR2^-^ and Ly6C^high^ CCR2^+^ macrophages in response to TAC in the myocardium. Notably, in sham animals, there was already a disparity in the number of Ly6C^low^ macrophages in the LV tissue, indicating significantly fewer Ly6C^low^ macrophages in Ccr2^-/-^ mice compared to Wt mice. Furthermore, at 3 and 6 days post-TAC, the number of Ly6C^low^ macrophages significantly increased in Wt mice compared to that of sham, whereas the difference reached significance only at day 3 post-TAC in *Ccr2*^*-/-*^ mice compared to the sham group (Fig 3C). As for the kinetics of Ly6C^high^ macrophages, we observed a significant increase in the Wt group at day 6 compared to sham. Additionally, the number of Ly6C^high^ macrophages was significantly higher at days 3 and 6 after TAC compared to *Ccr2*^*-/-*^ mice. Moreover, the number of Ly6C^high^ macrophages decreased in the LV myocardium following TAC in the *Ccr2*^*-/-*^ group (Fig 3D).

### A lack of CCR2 leads to a significant reduction in CD11b fluorescence intensity on Ly6C^high^ macrophages

In the subsequent phase, we assessed myocardial macrophage activation by measuring CD11b Mean Fluorescence Intensity (MFI). CD11b, a type 1 transmembrane glycoprotein, has been demonstrated to play a crucial role in mediating macrophage adhesion, migration, chemotaxis, and accumulation during inflammation [19]. Concentrating on the CD11b MFI of cardiac Ly6C^low^ macrophages, we observed a notable increase in fluorescence intensity at days 3 and 6 after TAC in the Wt groups compared to sham. Conversely, in CCR2-deficient mice, CD11b MFI increased significantly only at day 3 following TAC compared to sham (Fig 3E). Notably, the population of Ly6C^high^ macrophages exhibited markedly higher MFI in the sham group compared to Ly6C^low^ macrophages. Remarkably, we observed highly significant disparities in the fluorescence intensity of CD11b when comparing Ly6C^high^ macrophages of Wt and CCR2-deficient mice across the different groups. Specifically, the CD11b MFI of Ly6C^high^ macrophages from *Ccr2*^*-/-*^ sham mice was nearly 29 times lower compared to the MFI measured on the surface of Wt macrophages in the sham group (Fig 3F).

In summary, the innate immune response, with a specific focus on cardiac macrophages, significantly differs between Wt and CCR2-deficient mice, indicating that CCR2-CCL2 signaling contributes to the development of LV hypertrophy.

### IL-1β secretion is significantly reduced in response to TAC in CCR2 deficient mice

**Fig 4:**
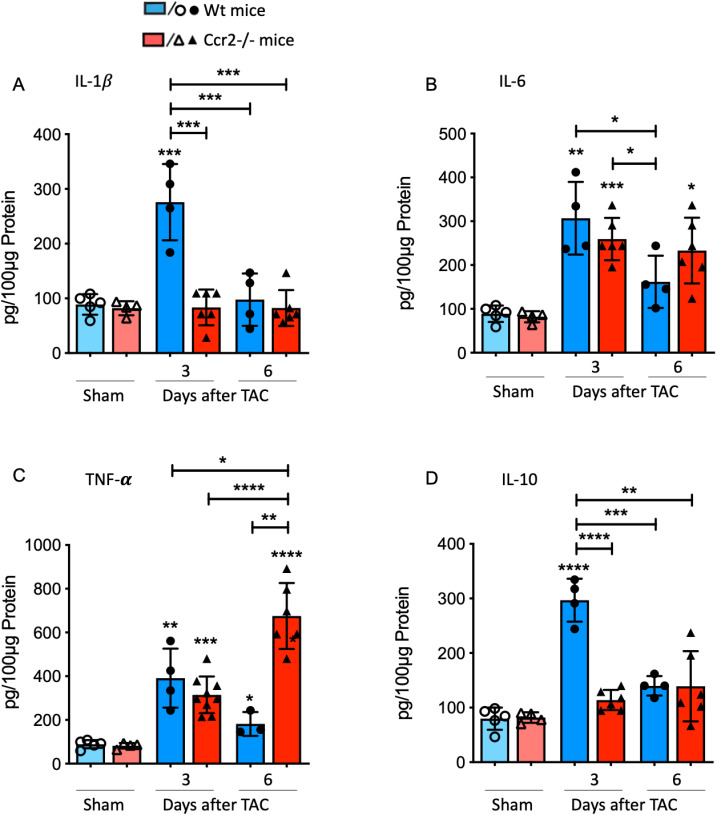
CCR2 deficiency modulates the cytokine pattern in the LV tissue in response to TAC. (A-**D)** Concentrations of the pro-inflammatory cytokines IL-1β, IL6, and TNF-α and the anti-inflammatory cytokine IL-10 were determined in LV tissue homogenates at day 3 and 6 after surgical intervention in *Ccr2*^*-/-*^ and Wt mice using ELISA assays. * above individual columns indicate significant differences between TAC and respective sham group; *P <0.05, **P<0.01, *** P<0.001, ****p < 0.0001.

The investigation of key cytokines implicated in cardiovascular disease pathogenesis is paramount, as it adds another crucial piece to the puzzle of understanding the immune response in LV hypertrophy development. In our study, impaired CCR2-CCL2 signaling resulted in hindered IL-1β secretion. Specifically, at day 3 post-TAC, the IL-1β level in LV tissue of Wt mice was significantly elevated compared to all other groups (Fig 4A). Furthermore, the protein concentration of IL-6 significantly increased in both Wt and *Ccr2*^*-/-*^ mice at day 3 post-TAC compared to their respective controls (Fig 4B). However, while the IL-6 level notably decreased in Wt mice between days 3 and 6, this decrease was less pronounced in CCR2-deficient mice. Consequently, the IL-6 concentration remained significantly elevated compared to controls in the *Ccr2*^*-/-*^ group, albeit slightly higher than the respective Wt group.

Tumor necrosis factor-alpha (TNF-α) is a proinflammatory cytokine produced by cardiac cells in response to stress. Elevated levels of TNF-α, both in circulation and the myocardium, are observed in dilated cardiomyopathy, myocardial infarction, and LV pressure overload, and are thought to contribute to ventricular remodeling [20,21]. In our study, we noted a significant increase in TNF-α concentrations in the myocardium at days 3 and 6 post-TAC in both, Wt and CCR2-deficient mice compared to their respective controls (Fig 4C). Interestingly, the level of TNF-α in *Ccr2*^*-/-*^ mice was notably higher at day 6 compared to day 3 and the Wt groups. Interleukin-10 (IL-10), a major anti-inflammatory cytokine expressed in the heart, may play a crucial role in cardiac remodeling. Evidence suggests that IL-10 potentially reduces pathological hypertrophy, hypothesized to occur through signaling via the IL-10 receptor (IL10R) in the heart, exerting a protective role in reducing cardiac hypertrophy [22]. Most inflammatory cells, including macrophages, can synthesize and secrete IL-10 [23]. In our study, we observed a significant increase in IL-10 levels in the myocardium of Wt animals at day 3 post-TAC compared to sham and all other groups (Fig 4D). However, in CCR2-deficient animals, no rise in IL-10 levels was evident in response to TAC.

The cytokine signature of *Ccr2*^*-/-*^ mice in LV hypertrophy development indicates a noteworthy reduction in IL-1β and IL-10 concentrations in the myocardium, while the production of IL-6 was not significantly altered, and the level of TNF-α further increased, peaking at day 6 compared to the cytokine response of CCR2-competent animals.

In summary, the cytokine pattern determined in CCR2 deficient mice indicates a less intense pro-inflammatory cytokine secretion at the early time-point day 3 after TAC compared to day 6.

## Discussion

Deficiency in CCR2 expression results in modifications to both the cellular and humoral immune responses following pressure overload induced by transverse aortic constriction. These changes potentially contribute to a cardioprotective mechanism, leading to reduced hypertrophy and BNP expression observed in CCR2-deficient mice subjected to TAC. Recent analyses have shown that the absence of the fractalkine receptor CX3CR1 results in a pro-inflammatory phenotype characterized by an increased presence of Ly6C^high^ macrophages in the myocardium, attributable to chemokine receptor CCR2-dependent monocyte recruitment from the circulation and overall depicting a cardioprotective effect [6]. In this study, we examined the role of CCR2 in the development of left ventricular hypertrophy using *Ccr2*^*-/-*^ mice.

Our findings underscore notable alterations in the cellular immune response observed in *Ccr2*^*-/-*^ *mice*, which may contribute to the observed attenuation of hypertrophy development and decreased BNP expression, a biomarker of cardiac dysfunction, in response to pressure overload. In contrast to our investigations conducted in CX3CR1-deficient mice in which no hypertrophy could be detected, CCR2 ko mice exhibited a HW/BW-Index on day 3 that did not reach statistical significance when compared to control mice but increased to significant hypertrophy at day 6. Coupled with the markedly reduced expression of BNP in the myocardium of CCR2 mice relative to Wt mice after TAC, this observation suggests a preservation of cardiac function in the absence of CCR2 on day 3.

Neutrophils and TNF-α secretion were notably higher on day 6 in CCR2 ko mice compared to Wt animals, while at the early time-point of day 3, we observed reduced pro-inflammatory activity in comparison with wild-type animals. The IL-1β concentration in the myocardium of *Ccr2*^*-/-*^ TAC mice did not surpass the levels observed in control mice at any time point. Those results align with our hypothesis that early inflammation, driven by pro-inflammatory immune cells, is advantageous for preserving myocardial integrity and the lack of pro-inflammatory CCR2^+^ Ly6C^high^ macrophages has a protective effect. Building on our recent findings in CX3CR1-deficient mice, we postulate that early pro-inflammation may confer a cardioprotective effect in the development of left ventricular hypertrophy due to pressure overload [6]. The MCP-1/CCR2 axis plays a pivotal role in directing monocyte migration towards sites of inflammation, acting as a crucial mediator in various pathological conditions such as atherosclerosis and cancer. This chemokine-receptor interaction not only facilitates monocyte recruitment but also amplifies the inflammatory response by activating downstream signaling pathways that promote the release of additional pro-inflammatory mediators [24].

In summary, our observations in CCR2-deficient mice reveal a delayed pro-inflammatory immune response characterized by reduced macrophage numbers and later neutrophil accumulation.

Neutrophils are known to serve as crucial first responders in the innate immune system following myocardial infarction, where they initiate inflammation to clear dead cell debris; however, their excessive accumulation can lead to myocardial injury through the release of harmful substances. Recent studies indicate that neutrophils not only have detrimental effects, such as forming extracellular traps (NETs) and producing extracellular vesicles (EVs) that exacerbate inflammation, but they also play beneficial roles in promoting anti-inflammatory responses, angiogenesis, and cardiac repair during post-MI remodeling [25]. Our findings highlight a significant increase in neutrophils in the myocardial tissue at day 6 after TAC induction that correlates with the increase in TNF-α levels in the CCR2 deficient mice. This observation might be due to the lack in early increase of IL1- β or other chemotactic substances that hamper the recruitment of neutrophils and the intense release of TNF-α by these cells.

Within the bone marrow, Ly^6^C^high^ monocytes originate from CCR2^+^ hematopoietic stem cells, progressing through sequential developmental stages involving common myeloid progenitors, and ultimately, committed monocyte progenitors [26]. This process has been reported to be regulated by the action of IL-1β released from the myocardium, which acts on bone marrow stem cells [27]. Our data highlights a lack of IL-1β secretion in CCR2-deficient mice compared to Wt animals and controls in response to TAC at day 3. This observation can be attributed to the absence of Ly6C^high^ macrophages in the myocardium, which are known sources of IL-1β in the orchestration of the cardiac immune response [28].

Regarding the transgenic mouse strain used in this study, we observed the expected significantly lower numbers of Ly6C^high^ macrophages in the myocardium compared to Wt animals. This could be attributed to the absence of chemotaxis via CCR2 to the site of inflammation. Interestingly, the absence of CCR2 has been associated with attenuated fibrosis, as CCR2^+^ macrophages are critical mediators in the fibrotic response by promoting fibroblast activation and extracellular matrix deposition [29]. This reduced fibrotic response may contribute to the observed preservation of left ventricular function and decreased hypertrophy in CCR2-deficient mice [30].

As our focus was the systematic analysis of the innate immune response, we did not put consideration to T cell immunology in our transgenic mouse model. We are aware of the fact that the chemokine CCL17, expressed by CCR2^+^ macrophages and dendritic cells, inhibits the recruitment of Tregs to the heart and the use of CCR2 deficient mice might influence T cell response in our model [31]. In LV hypertrophy, Tregs play a protective role by regulating the immune response and suppressing inflammation which might contribute to the immune-suppressive effect of the reduced number of inflammatory monocytes and macrophages in our model. Further studies are needed to shed light on this aspect [32].

In recently published studies, we have already demonstrated that the majority of macrophages in the myocardium in response to TAC are recruited via CCL2-CCR2 on the surface of monocytes/macrophages [6]. In addition to the reduced accumulation of Ly6C^high^ macrophages in the tissue of CCR2-deficient mice, our data highlights a significantly lower number of Ly6C^low^ macrophages in the LV at baseline levels, as well as at both time points following TAC in these animals, indicating an influence of CCR2 signaling on this macrophage population. We attribute this phenomenon to the known possibility of circulating monocytes differentiating into Ly6C^low^ macrophages in the tissue. The conversion of Ly6C^high^ monocytes/macrophages into Ly6C^low^ macrophages through phenotypic switching is recognized as an important source of tissue macrophages [33].

The assessment of the activation marker CD11b through fluorescence intensity detection revealed a significantly reduced mean fluorescence intensity (MFI) on the surface of Ly6C^high^ macrophages in *Ccr2*^*-/-*^ mice compared to Wt animals. We hypothesize that the diminished number of Ly6C^high^ macrophages in the LV tissue may exhibit lower activation, possibly due to a deficiency in IL-1β or CCL2 signaling, resulting in reduced CD11b expression levels. When examining the CD11b MFI of Ly6C^low^ macrophages, we did not observe significant differences between CCR2 competent and deficient mice, suggesting that the level of activity was not influenced by the absence of CCR2.

Ly6C^high^ macrophages are known to secrete pro-inflammatory cytokines such as IL-1β, IL-6, and TNF-α. Our findings emphasize that the absence of inflammatory Ly6C^high^ macrophages, resulting from disrupted CCR2 signaling, alters the cytokine profile in the myocardium following TAC intervention. Ly6C^low^ macrophages produce cytokines, including IL-10, which play a role in collagen formation and tissue repair [34]. The concentration of IL-10 did not increase in CCR2-deficient mice in response to TAC, whereas in CCR2-competent animals, a significant increase was detected. This observation may correlate with the reduced number of Ly6C^low^ macrophages in the myocardium of CCR2-deficient animals following TAC.

## Conclusions

In summary, our findings demonstrate that CCR2-dependent recruited macrophages play a role in the development of left ventricular hypertrophy (LVH). The early, less intense pro-inflammatory immune response may contribute to the attenuation of hypertrophy development and BNP expression, suggesting a cardioprotective effect of early pro-inflammation in the context of pressure overload-induced heart damage.

## Supporting information

Supplementary Table 1 Minimal data set for **figures 2**–**4**.
(PDF)
