# Supplementary material for: CCR2 dependent recruited pro-inflammatory monocytes contribute to the development of left ventricular hypertrophy in mice upon transverse aortic constriction
Source: PLoS One. 2025 Apr 21;20(4):e0318407. doi: 10.1371/journal.pone.0318407 (PMC12011267; doi:10.1371/journal.pone.0318407)
Supplement: Supplementary Table 1 — Minimal data set for figures 2–4. (PDF) [file pone.0318407.s001.pdf]

Minimal data set for figures 2, 3 and 4

Figure #		Mean	SD	S.E.M.	Statistical method used	P value	# samples
**2A**							
Sham Wt	5,7<br>4,6<br>5,5<br>5,2<br>5,0<br>5,4	5.233	0.3933	0.1606	Shapiro-Wilks normality test, t-test, One-Way-Anova	*P <0.05, **P<0.01, *** P<0.001, ****p < 0.0001.	6
Sham Ccr2-/-	4,9<br>5,1<br>5,3<br>4,7<br>5,1<br>4,8	4.983	0.2229	0.09098			6
3d TAC Wt	7,0<br>5,4<br>5,6<br>6,1<br>6,0<br>5,9	6.000	0.5550	0.2266			6
3d TAC Ccr2-/-	4,8<br>5,2<br>5,6<br>6,2<br>5,1<br>5,3	5.364	0.4850	0.1980			6
6d TAC Wt	7,4<br>8,3<br>6,6<br>6,2<br>7,6<br>6,8	7.150	0.7635	0.3117			6
6d TAC Ccr2-/-	5,3<br>6,4<br>5,7<br>5,2<br>5,6<br>6,1	5.717	0.4622	0.1887			6

**2B**							
3d TAC Wt	58,068<br>58,068<br>58,068<br>63,441<br>63,441<br>63,441	60.75	2.943	1.201	Shapiro-Wilks normality test, t-test, One-Way-Anova	*P <0.05, **P<0.01, *** P<0.001, ****p < 0.0001.	6
3d TAC Ccr2-/-	58,906<br>58,906<br>58,906<br>38,249<br>38,249<br>38,249<br>29,48<br>29,48<br>29,48<br>59,384<br>59,384<br>59,384<br>44,416<br>44,416<br>44,416<br>30,541<br>30,541<br>30,541<br>33,405<br>33,405<br>33,405	42.05	12.06	2.632			21
6d TAC Wt	71,907<br>71,907<br>71,907<br>60,605<br>60,605<br>60,605<br>86,041<br>86,041<br>86,041	72.85	11.04	3.679			12
6d TAC Ccr2-/-	58,337<br>58,337<br>58,337<br>90,03<br>90,03<br>90,03<br>47,637	57.50	21.59	6.232			9

	47,637<br>47,637<br>33,989<br>33,989<br>33,989						
**3A**							
Sham Wt	7617,03<br>7493,64<br>8342,01<br>18310,19<br>15113,68<br>8025,22	10817	4686	1913	Shapiro-Wilks normality test, t-test, One-Way-Anova	*P <0.05, **P<0.01, *** P<0.001, ****p < 0.0001.	6
Sham Ccr2-/-	14407,42<br>19626,12<br>7734,91<br>8327,37<br>11087,54<br>12062,84	12208	4387	1791			6
3d TAC Wt	27324,23<br>19871,07<br>22627,74<br>27888,91<br>19475,48<br>14671,3	21976	5060	2066			6
3d TAC Ccr2-/-	16531,731<br>20945,44<br>26633,69<br>10228,11<br>23665,15<br>21406,4	19902	5793	2365			6
6d TAC Wt	12651,61<br>3349,79<br>4271,42<br>8868,74<br>3433,35<br>13620,86	7699	4687	1913			6
6d TAC Ccr2-/-	40010,74<br>30476,51<br>28718,67<br>41785,99<br>24005,39<br>26930,38	31988	7248	2959			6
**3B**							

Sham Wt	34999,85<br>43313,35<br>33710,71<br>14663,65<br>13631,45<br>18299,3	26436	12489	5099	Shapiro-Wilks normality test, t-test and non-parametric test, One-Way-Anova and non-parametric test	*P <0.05, **P<0.01, *** P<0.001, ****p < 0.0001.	6
Sham Ccr2-/-	98756,13<br>99785,02<br>88560,46<br>68349,64<br>72289,33<br>84500,67	20262	3546	1448			6
3d TAC Wt	68777,31<br>52825,8<br>68804,96<br>59427,97<br>32796,66<br>72804,96	59240	1487	6074			6
3d TAC Ccr2-/-	27579,97<br>29186,35<br>14096,6<br>29594,94<br>10793,96<br>28250,89	23250	8464	3455			6
6d TAC Wt	175339,5<br>213976,9<br>250249,3<br>177979,5<br>228331,9<br>264707,3	218431	36783	15017			6
6d TAC Ccr2-/-	98756,13<br>99785,02<br>88560,46<br>68349,64<br>72289,33<br>84500,67	85374	13105	5350			6
**3C**							
Sham Wt	29476,49<br>60994,83<br>35984<br>42378<br>31105,22<br>27234,86	37862	12554	5125	Shapiro-Wilks normality test, t-test and non-parametric test, One-Way-Anova	*P <0.05, **P<0.01, *** P<0.001, ****p < 0.0001.	6
Sham Ccr2-/-	8361,93	14852	5221	2132			6

	15593<br>17816,25<br>18633,82<br>20272,9<br>8435,77				and non-parametric test		
3d TAC Wt	105005,5<br>212925,8<br>114434,9<br>83661,28<br>108271,6<br>119868,3	124028	45281	18486			6
3d TAC Ccr2-/-	22499,2<br>17075,53<br>46680,71<br>28651,16<br>24142,98<br>24404,82	27242	10231	4177			6
6d TAC Wt	165604,5<br>105544,7<br>93964,82<br>204681,9<br>178127,3<br>234391,8	163719	55016	22460			6
6d TAC Ccr2-/-	7485,37<br>8352,89<br>16133,07<br>10992,35<br>11591,28<br>17771,22	12054	4129	1686			6
**3D**							
Sham Wt	3686,61<br>4391,45<br>3590,33<br>3573,45<br>3513,68<br>1209,66	3328	1087	443.9	Shapiro-Wilks normality test, t-test and non-parametric test, One-Way-Anova and non-parametric test	*P <0.05, **P<0.01, *** P<0.001, ****p < 0.0001.	6
Sham Ccr2-/-	2020,45<br>1241,36<br>2856,07<br>4462<br>2415,65<br>2628,14	2604	1072	437.8			6
3d TAC Wt	5292,98<br>3573,45	4170	1554	634.5			6

	4000,59<br>2413,56<br>3083,22<br>6653,27						
3d TAC Ccr2-/-	530,86<br>1326,92<br>815,69<br>2022,29<br>861,88<br>1559,13	1186	553.1	225.8			6
6d TAC Wt	7917,74<br>4266,86<br>4669,56<br>5268,62<br>8518,82<br>4270,09	5819	1904	777.3			6
6d TAC Ccr2-/-	1040,81<br>1487,25<br>1885,1<br>2296,76<br>1214,79<br>1809,98	1622	465.1	189.9			6
**3E**							
Sham Wt	4554<br>1783<br>3582<br>4705<br>2807<br>4208	3607	1134	463.0	Shapiro-Wilks normality test, t-test and non-parametric test, One-Way-Anova and non-parametric test	*P <0.05, **P<0.01, *** P<0.001, ****p < 0.0001.	6
Sham Ccr2-/-	5415<br>5291<br>6269<br>4660<br>6644<br>4169	5408	935.7	382.0			6
3d TAC Wt	7753<br>4041<br>2961<br>6788<br>5485<br>3971	5167	1843	752.3			6
3d TAC Ccr2-/-	8116<br>7637<br>5633	7213	858.3	350.4			6

	7342<br>6984<br>7563						
6d TAC Wt	6003<br>5976<br>7219<br>8100<br>3479<br>7534	6385	1656	676.1			6
6d TAC Ccr2-/-	6938<br>5600<br>5879<br>7780<br>6327<br>6107	6439	798.4	326			6
**3F**							
Sham Wt	84167<br>76780<br>80403<br>40244<br>97822<br>57285	72784	20638	8425	Shapiro-Wilks normality test, t-test and non-parametric test, One-Way-Anova and non-parametric test	*P <0.05, **P<0.01, *** P<0.001, ****p < 0.0001.	6
Sham Ccr2-/-	2575<br>2810<br>1833<br>3089<br>2662<br>2228	2533	444.4	181.4			6
3d TAC Wt	67069<br>44480<br>24539<br>64194	50071	19761	9881			4
3d TAC Ccr2-/-	3097<br>5037<br>2695<br>4681<br>3847<br>4627	3997	945.9	386.2			6
6d TAC Wt	71271<br>62706<br>36604<br>57927<br>75881<br>65150	61590	13781	5626			6

6d TAC Ccr2-/-	2000<br>1826<br>1720<br>2979<br>2742<br>1764	2172	547	223.3			6
**4A**							
Sham Wt	92,17<br>108,29<br>84,93<br>59,00<br>99,87	88.86	18.82	8.47	Shapiro-Wilks normality test, t-test and non-parametric test, One-Way-Anova and non-parametric test	*P <0.05, **P<0.01, *** P<0.001, ****p < 0.0001.	5
Sham Ccr2-/-	83,87<br>86,88<br>63,87<br>93,87	82.12	12.87	6.435			4
3d TAC Wt	183,87<br>308,88<br>264,87<br>345,87<br>6,81<br>11,44<br>9,81<br>12,81	275.9	69.70	34.85			4
3d TAC Ccr2-/-	109,62<br>71,55<br>107,62<br>28,62<br>110,60<br>71,55	83.43	32.69	13.35			6
6d TAC Wt	128,25<br>146,88<br>44,55<br>71,55	97.81	47.82	23.91			4
6d TAC Ccr2-/-	71,55<br>82,62<br>65,88<br>55,62<br>71,55<br>146,88	82.35	32.81	13.40			6
**4B**							
Sham Wt	72,78<br>113,00<br>99,13	99.26	15.95	7.13	Shapiro-Wilks normality test, t-	*P <0.05, **P<0.01, ***	5

	110,39<br>100,98				test and non-parametric test, One-Way-Anova and non-parametric test	P<0.001, ****p < 0.0001.	
Sham Ccr2-/-	99,61<br>118,95<br>132,47<br>121,18	118.1	13.65	6.82			4
3d TAC Wt	334,53<br>244,13<br>412,02<br>237,033	306.9	82.93	41.47			4
3d TAC Ccr2-/-	195,80<br>291,03<br>336,74<br>246,15<br>244,14<br>242,13	259.3	48.43	19.77			6
6d TAC Wt	145,53<br>244,13<br>155,52<br>102,033	161.8	59.60	29.80			4
6d TAC Ccr2-/-	336,74<br>195,80<br>291,03<br>244,13<br>124,07<br>207,52	233	75.04	30.63			6
**4C**							
Sham Wt	98,58<br>46,61<br>84,47<br>93,01<br>76,63	79.86	20.38	9.11	Shapiro-Wilks normality test, t-test and non-parametric test, One-Way-Anova and non-parametric test	*P <0.05, **P<0.01, *** P<0.001, ****p < 0.0001.	5
Sham Ccr2-/-	77,99<br>89,13<br>70,13<br>90,13	81.85	9.55	4.78			4
3d TAC Wt	334,53<br>244,13<br>317,52<br>291,03	296.8	39.41	19.71			4
3d TAC Ccr2-/-	117,99<br>106,65<br>140,13<br>129,33	113.9	18.59	7.59			6

	94,77<br>94,77						
6d TAC Wt	140,13<br>117,99<br>161,73<br>140,13	140	17.86	8.93			4
6d TAC Ccr2-/-	66,74<br>195,80<br>102,03<br>109,13<br>124,07<br>237,52	139.2	64.24	26.23			6
**4D**							
Sham Wt	97,63<br>111,10<br>104,00<br>65,52<br>103,60	96.37	17.89	8.0	Shapiro-Wilks normality test, t-test and non-parametric test, One-Way-Anova and non-parametric test	*P <0.05, **P<0.01, *** P<0.001, ****p < 0.0001.	5
Sham Ccr2-/-	151,00<br>97,00<br>109,01<br>98,02	113.8	25.42	12.71			4
3d TAC Wt	334,53<br>244,13<br>425,52<br>561,033	391.3	135.2	67.61			4
3d TAC Ccr2-/-	270,00<br>297,01<br>297,01<br>216,00<br>324,00<br>378,00<br>480,6<br>214,65<br>357,21	314.9	83.52	27.84			6
6d TAC Wt	145,53<br>244,13<br>155,52	181.7	54.28	31.34			3
6d TAC Ccr2-/-	892,08<br>797,31<br>697,95<br>480,60<br>592,92<br>592,92	675.6	150.9	61.61			6
